# Adaptation of a small animal radiation research platform for pre‐clinical microbeam irradiations

**DOI:** 10.1002/mp.70162

**Published:** 2025-11-25

**Authors:** Lauren Yeomans, Kimia Gharehkhani, Craig Cummings, Joanna Wilk, Carol Box, Simeon Nill, Uwe Oelfke

**Affiliations:** ^1^ Division of Radiotherapy and Imaging, The Institute of Cancer Research, London, UK and Joint Department of Physics The Royal Marsden NHS Foundation Trust London UK

**Keywords:** microbeam, radiotherapy, spatial fractionation

## Abstract

**Background:**

Microbeam radiotherapy (MRT) was introduced as a method to widen the therapeutic window compared to standard radiotherapy treatments. Studies have shown the normal tissue sparing effects of MRT, whilst maintaining the tumor control of standard broad beam radiotherapy.

**Purpose:**

To create and test a microbeam (MB) delivery system compatible with a small animal radiation research platform (SARRP, Xstrahl), which would allow the machine to be easily adapted to deliver MB irradiations without making any permanent changes to the system.

**Methods:**

A MB collimator was designed, that fits in with the existing SARRP infrastructure and can easily be removed to revert the machine back to a normal broad beam delivery system. A translatable mousebed was developed to reduce the source‐to‐surface distance (SSD) for MB delivery, allowing the target position to be moved between imaging and treatment. Gafchromic film was used to test the accuracy of the positioning, and to determine the dose rates achievable in a solid water phantom.

**Results:**

At 1 mm depth in solid water, a peak‐to‐valley dose ratio (PVDR) of 46 was achieved when the target was placed directly under the collimator, whilst a PVDR of 29 was reached when a 10.5 mm air gap was introduced between the collimator and target. Integral dose rates at 1 mm depth for these setups were 0.93 ± 0.04 and 0.94 ± 0.01 Gy/min, respectively. Conversion between broad beam and microbeam setup was found to take less than 5 min.

**Conclusions:**

Our adaptations enable any SARRP machine to be converted into a MB delivery system, with feasibility studies confirming MB delivery in a solid water phantom. The system is now routinely used to deliver MBs for in vivo studies.

## 1 INTRODUCTION

Spatially fractionated radiotherapy (SFRT) delivers radiation in modulated patterns rather than uniformly, with microbeam radiation therapy (MRT) being one of the most widely studied pre‐clinical approaches. MRT employs arrays of micrometre‐wide x‐ray beams consisting of high‐dose peaks and low‐dose valleys, producing a distinctive dose distribution that has been shown to spare normal tissues while maintaining tumor control. Synchrotron‐based MRT studies typically use beam widths of 25–75 µm with center‐to‐center (CTC) distances of 100–400 µm, yielding peak entrance doses of several hundred Gy and peak‐to‐valley dose ratios (PVDRs) of 20–40 at shallow depths.^1^, ^2^

Normal tissues can tolerate peak MRT doses far above conventional homogeneous doses; however, current evidence suggests that valley dose, rather than peak dose, is more strongly linked to normal tissue response, and there is still no consensus on which parameter (peak, valley, integral dose, PVDR, or beam spacing) best predicts tumor control or tissue sparing.^2^, ^3^ A few studies have included in vivo central nervous system (CNS) models that test whether microbeam (MB) arrays can spare dose‐limiting organs‐at‐risk, leveraging MRT's preferential normal‐tissue sparing. These studies demonstrate that normal tissues exhibit rapid repair, supported by minimally irradiated cells adjacent to the MB paths.^4^

Although synchrotrons remain the gold standard for MRT, due to their minimal divergence and high dose rates, their limited accessibility has driven the development of compact irradiators. Monte Carlo studies and recent experimental work with small animal irradiators, such as the SARRP and XenX, have demonstrated that slit widths of ∼100–200 µm and PVDRs of 15–40 can be achieved at clinically relevant depths with a small focal spot, albeit with lower dose rates (0.5–1 Gy/min),^1^, ^5^ thereby broadening access to MRT research for biological and translational studies.

## 2 METHODS

The small animal radiation research platform (SARRP) is a pre‐clinical radiation platform created by Xstrahl (Walsall, UK), consisting of an industrial Varian NDI‐225‐22 stationary anode x‐ray tube (Varian Medical Systems, Salt Lake City, USA) within a fully shielded cabinet. It provides a homogeneous 220 kVp x‐ray field of variable size, depending on the collimator used. Collimators are inserted and removed on precision v‐rails. All irradiations discussed here were performed with the maximum current of 13.0 mA, broad focal spot size of 5.5 mm (per EN12543), inherent filtration of 0.8 mm ± 0.1 mm beryllium, and additional filtration of 0.15 mm copper.

The MB collimator consists of three tungsten plates with a combined thickness of 7 mm (outer plates 1.75 mm each, central plate 3.5 mm), adapted from a design described by Treibel et al. (2021).^6^ This design allows for the MB peak width to be adjusted by moving the central plate. The tungsten plates were mounted in an aluminium frame, attached to a collimator head with four rods. The top of the collimator slides into the existing v‐rail mechanism of the SARRP treatment head.

MB slits in the tungsten plates were made by electrical discharge machining by FEOB GMBH (Forstern, Germany). Each slit has a maximum width of 170 µm with a manufacturing tolerance of ± 5 µm, and a CTC of 400 µm. A total of 51 slits make up a MB field size at the exit of the collimator of 20 mm x 20 mm. Slits were angled to account for the divergent x‐ray source, with the angles computed for a source to collimator distance of 21 cm, where the beam is homogeneous over the required 20 mm x 20 mm field.

The position of the central plate of the collimator is controlled by a Piezo Inertia actuator (PIAK10, Thorlabs Inc., Newton, USA) which has a total travel distance of 10 mm and typical step‐size of 20 nm. On the opposite side of the central plate are two springs and a stylus‐type high‐precision sensor head (GT2‐P12K, Keyence, Neuherberg, Germany), which has a total measuring range of 12 mm and a resolution of 0.1 µm. The actuator is controlled via a MATLAB application (Version R2022b, Mathworks, Natick, USA) which also provides the sensor readout. The sensor position provides an arbitrary number based on a linear scale. The sensor is initialised each session by moving the sensor against a physical hard stop. This position is then defined as the origin. From this position, the sensor is then moved to the value determined to provide the required physical slit width. The sensor position is always approached from one direction to avoid any backlash in the system.

The normal isocentre of the SARRP is at 35 cm from the source. Given the reduction in dose rate from the photons absorbed in the tungsten collimator (less than 20% transmission through the open slits), the source‐to‐surface distance (SSD) must be reduced for MB delivery to increase the dose rate at the target. However, to use the computed tomography (CT) imaging for tumor targeting, the animal must be placed at the normal isocentre. For this reason, an Xstrahl mousebed was modified so that it could be used for imaging at the normal isocentre then elevated to reduce the SSD for treatment.

**FIGURE 1:**
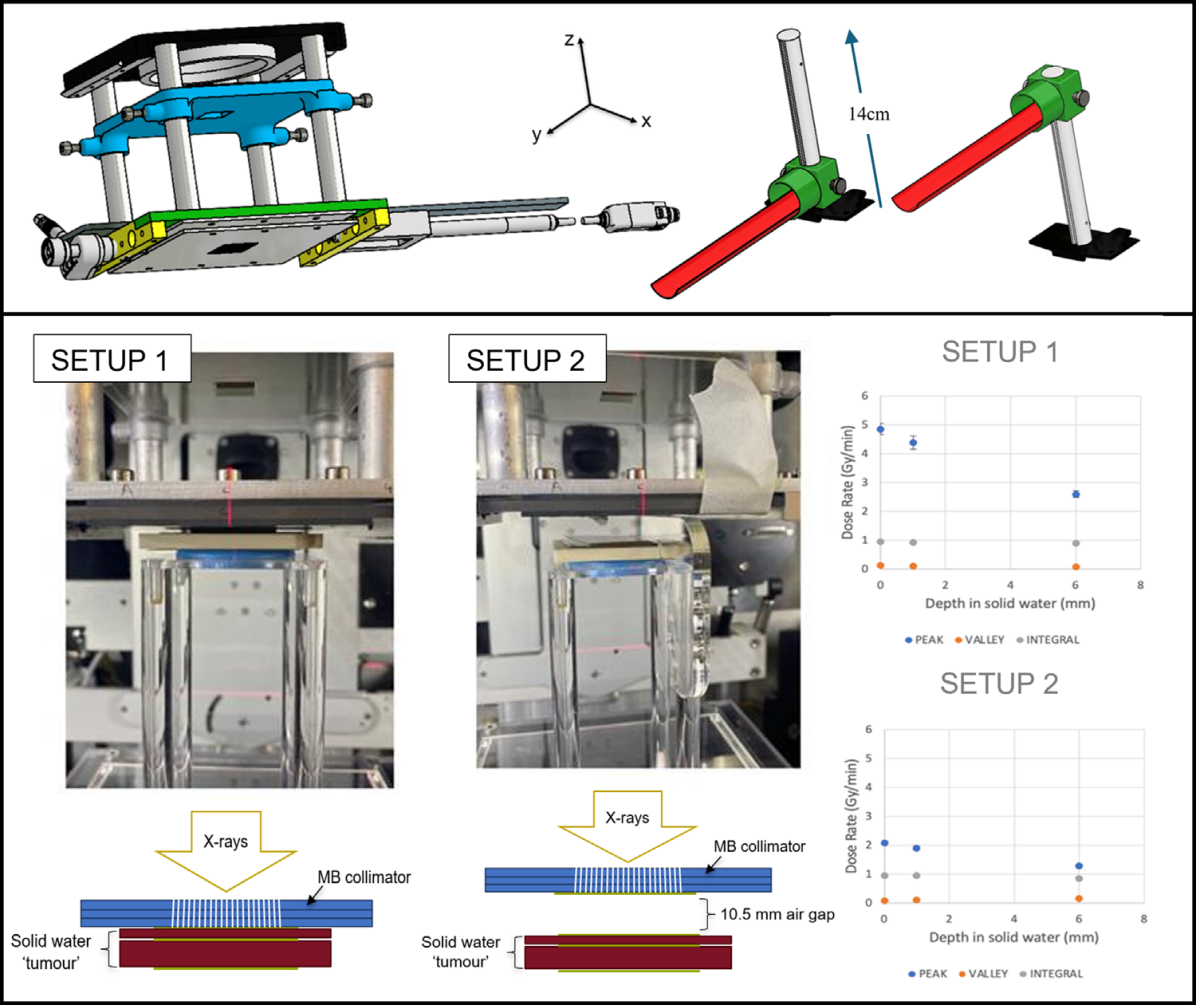
Top left: A drawing of the collimator assembly, showing the pre‐collimator in blue, actuator on the left‐hand side, and sensor on the right‐hand side. Top right: The adapted mousebed shown at imaging (left) and treatment (right) positions, allowing the target to be raised from the normal isocentre (SSD = 35 cm) to treatment plane (SSD = 21 cm). Bottom left: The two setups investigated using a solid water phantom, with film at the surface, 1 mm depth and 6 mm depth. Bottom right: Peak, valley and integral dose rates in depth for the two setups shown.

Performing the CT imaging using the SARRP software enables the target to be centred in the (*x*, *y*) plane (see Figure 1 for coordinate system) using MuriPlan (treatment planning software, Xstrahl).

Tests conducted to assess the *x–y* accuracy of the translatable mousebed were performed using EBT‐XD Gafchromic film (Ashland Advance Materials, Bridgewater, USA), and an aluminum ball representing the target. The aluminum ball was placed on top of the film and targeted using Muriplan, using a multi‐variable collimator set to 20 mm x 20 mm. The film was irradiated at isocentre, then the mousebed was elevated to treatment position and the same film was irradiated again. The offset of the centre of the ball to the centre of the radiation field was assessed to test the presence of any *x–y* motion in the mousebed.

Figure 1 (top left) shows the full assembly of the MB collimator system including an additional (optional) pre‐collimator shown in blue which was added to reduce the overall field size entering the MB collimator. It consists of a holder (3D printed from MED610) supporting a 6 mm deep lead plate, with a 6 mm x 6 mm aperture to allow the photon beam through. The *z*‐position of the pre‐collimator was adjusted incrementally until the desired 10 mm x 10 mm field size at MB collimator exit was achieved. To perform field size measurements, film was placed at the MB collimator exit then scanned 48 h later, on a Zeiss Axioscan scanning microscope (Carl Zeiss Microscopy GmbH, Jena, Germany) using a 10X objective. Measurements were performed in ZEN (Version 3.2, blue edition) from the edges of the visible field.

To set the absolute width of the MBs, charge output through the collimator was measured using a Roos Electron Chamber (200 V, PTW 34001), and a Unidos E dosemeter (PTW, Freiburg, Germany) was used to read collected charge. The central plate was driven forwards using the actuator, and charge collected over a 60‐s interval was recorded for incremental sensor positions. The relationship between charge and sensor position was later used to set the position for the maximum MB slit width, assuming the maximum integral charge output is when tungsten plates are fully aligned. Slit widths were then confirmed for each position by measuring the full width half maxima (FWHM) of the five central peaks on the 2D dose profile. To do this, three films were exposed for each sensor position under investigation, resetting the system between each irradiation to ensure repeatability and reproducibility over time. Sensor positions were calculated for MB slit widths of 100 µm and the maximum of 170 µm. Furthermore, all dosimetry in this study was performed with the 100 µm slits, as shown in the results.

Film dosimetry was calibrated in a 20 cm × 20 cm solid water stack at 2 cm depth and an SSD of 35 cm, using a PTW 31010, (0.125cc) Semiflex ionisation chamber with a Unidos E dosemeter in a broad beam field, following the IPEMB code of practice.^7^ A calibration curve was generated from 20 films irradiated between 0 and 40 Gy, covering the full dynamic range of the measurements. For the MB dosimetry, films were scanned 48 h after irradiation (as described above) at a resolution of 0.08 mm/pixel, and images were analyzed in FilmQAPro V7 (Ashland Inc., Wilmington, USA), using the red color channel which was found to provide the best dose discrimination^8^ for both high (peak) and low (valley) doses.

Film orientation was kept consistent for all irradiations and scans. To minimize sensitivity to pixel‐level noise, dose values were averaged across a 4‐pixel width. Peak doses were defined as the average over the five central peaks of the maxima of the 2D dose profile perpendicular to the MB stripes. Valley doses were taken as the average of the five central minima. Integral dose is defined as the average dose over a circular region of interest (ROI) in the centre of the MB field, incorporating at least five MB stripes. No smoothing or post‐processing was applied to the raw data to preserve the integrity of the measurements, ensuring that the reported PVDR values represent the true physical measurements without bias from post‐processing.

A 60 mm × 60 mm × 5 mm (*x, y, z*) solid water phantom was used to represent a tumor target for dosimetry. Film was placed at the phantom surface, 1 mm depth, and 6 mm depth (phantom exit). Two different setups were investigated, shown in Figure 1 (bottom left), with and without an air gap between the target and the collimator. Firstly, setup 1 shows the target placed directly under the collimator, which was evaluated in order to obtain the greatest PVDR, as high PVDRs have been reported to drive the efficacy of MB therapy.^3^ However, slight variations in tumor positions and mouse anatomies mean that this is not always possible for in vivo studies. For this reason, setup 2 was also included to assess the impact of a 10.5 mm air gap on dose rates.

## 3 RESULTS

By raising the mousebed post‐CT an SSD of 21 cm was achieved when the target is placed directly below the collimator. The *x* and *y* offsets of the mousebed elevation were found to be 0.74 ± 0.1 mm and 1.10 ± 0.2 mm, respectively, when the elevator was raised the full 14 cm from the normal isocentre to the MB treatment plane. The test using the aluminum ball representing the tumor target showed that the target is fully covered by the treatment region after elevation, so the mousebed was not adjusted further.

Using a linear fit on the resultant field size plotted against source to pre‐collimator distances, the position of the pre‐collimator which provides a field size of 10 mm x 10 mm at the treatment plane was determined as 31 mm from the top of the assembly rods. The pre‐collimator was then fixed at this position for all further dosimetry measurements.

For the slit width measurements, maximum output was achieved at a sensor position of 0.290, which was found to produce a slit width of 168.1 ± 2.0 µm, measured using film in air. This is within the manufacturing tolerance of the plates provided by the manufacturer, which was 170 ± 5.0 µm. For the intended slit widths of 100 µm (sensor position 0.222) repeatability tests measured slits of 97.7 ± 2.0 µm (using film in air) over all analyzed microbeams.

**FIGURE 2:**
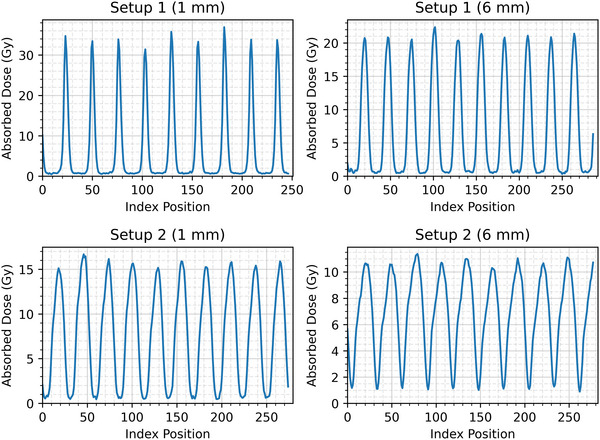
Dose profiles measured for setup 1 (no air gap) and setup 2 (with air gap) at 1 and 6 mm depths in the solid water phantom. Each profile corresponds to an irradiation time of 500 s with a 100 µm slit width.

Peak, valley, and integral dose rates (Gy/min) are shown in Figure 1 for the two solid water phantom setups described earlier, for MBs set to a slit width of 100 µm at the collimator. The introduction of an air gap decreased the PVDR at 1 mm from 46 to 29 (driven by the increase in valley dose) but resulted in no significant difference in the integral dose rates, being 0.93 ± 0.04 Gy/min (no air gap) and 0.94 ± 0.01 Gy/min (with air gap). FWHM measurements of the dose profiles yielded MB widths of 128.9 ± 2.3 µm and 331.5 ± 3.0 µm at phantom entry for setup 1 and 2, respectively. Conversion between broad beam and microbeam setup, including setting the slit width and replacing the mousebed, was found to take less than five minutes. Figure 2 shows the microbeam dose profiles for both setups. As expected, the peak dose decreased with depth and was lower in setup 2 due to the air gap. The valley dose increased slightly with depth, reflecting greater scatter contribution, resulting in reduced PVDR at 6 mm compared with 1 mm.

## 4 DISCUSSION

We have presented the first method for conversion of a SARRP for MB irradiations that can be easily reverted for conventional radiation delivery. Our results show PVDRs and dose rates comparable with those achieved on the XenX platform at the Helmholtz Centre for Environmental Health, (Munich, Germany),^5^ which also delivers microbeams without the use of a synchrotron.

Although we used the broad focal spot, the SARRP also has a fine focal spot and Monte Carlo simulations predicted that this would produce larger PVDRs^1^. Treibel et al. found that using the smaller focal spot on their XenX platform with 50 µm MB peak width enhanced PVDRs from 13 to 28 (at 1 mm depth) but reduced the peak dose rate by greater than 50%, from 2.71 to 1.17 Gy/min.^6^ When in vivo studies were performed with the same setup, the effective focal spot size was reduced again by placing the collimator at an angle to the source, further increasing the PVDR.^5^ However, due to the fine focal spot producing lower dose rates, and legal limitations (UK Home Office) on mouse treatment times, it was decided to proceed with the broad focal spot on the SARRP for our in vivo experiments.

Pre‐clinical MRT research will allow further investigation into the biological mechanisms underlying the ‘microbeam effect’. That said, the application is limited by the dose rates achievable with an x‐ray tube, with a maximum integral dose rate of around 1 Gy/min at 1 mm depth for our setup. Despite the dose maximisation techniques we have used, it is not possible with an x‐ray tube to match the dose rates achievable at synchrotron facilities such as ESRF, which can deliver 8–16 kGy/s^2^. However, the limited dose rate in our setup permits MB effects to be investigated in the absence of FLASH effects. Importantly, our 2D dose profiles (Figure 2) showed that the microbeam array maintained good spatial integrity, with peak doses varying by only ∼9% across the central ∼2 cm field and valley doses remaining stable. Thus, despite the lower dose rate, the uniformity and reproducibility of the array geometry remain sufficient to enable meaningful biological investigations.

In our setup, the introduction of a 10.5 mm air gap led to a reduction in PVDR, highlighting the limited physical treatment range. The highest PVDRs were achieved directly under the collimator, but tumor positioning at this location is not always feasible; therefore, initial in vivo experiments have focused on subcutaneous tumors that can be placed close to the collimator with good reproducibility between animals. The phantom was chosen to mimic a small tumor volume and to allow direct film placement at defined depths. While suitable for relative dosimetric comparisons (beam shaping, PVDR), this design does not fully capture scatter conditions at greater depths or in heterogeneous tissue.

One limitation of implementing MRT on conventional small‐animal irradiators is the prolonged irradiation time required to reach therapeutic doses, which contrasts with the ultrahigh dose rates achievable at synchrotron facilities. As reported in previous studies,^1^, ^5^ extended exposures can lead to blurring of the microbeam dose profiles due to physiological motion such as respiration, heartbeat, or vascular pulsations, thereby reducing the effective spatial resolution of the array, lowering PVDR, and compromising the distinct peak–valley structure that underpins the MRT effect. While our phantom‐based measurements were unaffected by motion, in vivo applications will require appropriate motion management strategies, including optimized anesthesia protocols, respiratory or cardiac gating, or focusing on anatomical sites with minimal motion such as the brain.

Using this method, our MB setup in the SARRP is now routinely used for in vivo experiments. Pilot studies have proved that MBs can safely and successfully be delivered to a mouse tumor, and we are now moving on to tumor growth studies to test the efficacy of MBs compared with conventional broad beam irradiations.

## 5 CONCLUSIONS

A MB collimator, compatible with a SARRP system has been designed and built. Additionally, a standard SARRP mousebed was adapted to elevate the treatment isocentre toward the x‐ray source so reducing the SSD. This yielded an increased dose rate enabling shorter treatment delivery times and more acceptable anaesthesia times for mice. A protocol for achieving a precise and reproducible slit width has been established and tested.

It has previously been shown that microbeams can be produced using a standard x‐ray tube. Our development of the mouse elevator and v‐rail collimator attachment means that MB treatments can set up quickly and require no permanent changes to the cabinet or additional shielding. The SARRP can be converted into a MB delivery system in less than 5 min and reverted to broad beam delivery just as easily.

## CONFLICT OF INTEREST STATEMENT

The authors declare no conflicts of interest.

## Data Availability

Authors will share data upon request to the corresponding author.
